# Celiac disease serology and gut microbiome following protein pump inhibitor treatment: Erratum

**DOI:** 10.1097/MD.0000000000022947

**Published:** 2020-10-16

In the article, “Celiac disease serology and gut microbiome following protein pump inhibitor treatment”,^[1]^ which originally appeared in Volume 99, Issue 35 of *Medicine*, the title appeared incorrectly and has since been updated to “Celiac disease serology and gut microbiome following proton pump inhibitor treatment.” Protein pump has also been updated to proton pump throughout the article.
